# Loss of functional pRB is not a ubiquitous feature of B-cell malignancies

**DOI:** 10.1038/sj.bjc.6690408

**Published:** 1999-05

**Authors:** A J Sinclair, V Frost

**Affiliations:** School of Biological Sciences, University of Sussex, Brighton, E Sussex, BN1 9QG, UK

**Keywords:** retinoblastoma protein, cdkI, p16^INK4a^, tumour suppressor, Burkitt's lymphoma, Epstein–Barr virus

## Abstract

Human cancers frequently sustain genetic mutations that alter the function of their G1 cell cycle control check point. These include changes to the retinoblastoma gene and to the genes that regulate its phosphorylation, such as the cyclin-dependent kinase inhibitor p16^INK4a^. Altered expression of retinoblastoma protein (pRb) is associated with non-Hodgkin's lymphoma, particularly centroblastic and Burkitt's lymphomas. pRb is expressed in normal B-cells and its regulatory phosphorylation pathway is activated in response to a variety of stimuli. Since human B-lymphoma-derived cell lines are often used as in vitro model systems to analyse the downstream effects of signal transduction, we examined the functional status of pRb in a panel of human B-cell lines. We identified eleven cell lines which express the hyperphosphorylated forms of pRb. Furthermore, we suggest that the pRb protein appears to be functional in these cell lines. © 1999 Cancer Research Campaign


*British Journal of Cancer* (1999) **80**(5/6), 670–675
© 1999 Cancer Research Campaign
Article no. bjoc.1998.0408

### Loss of functional pRB is not a ubiquitous feature of B-cell malignancies

**AJ Sinclair and V Frost**

School of Biological Sciences, University of Sussex, Brighton, E Sussex BN1 9QG, UK

**Summary** Human cancers frequently sustain genetic mutations that alter the function of their G1 cell cycle control check point. These include changes to the retinoblastoma gene and to the genes that regulate its phosphorylation, such as the cyclin-dependent kinase inhibitor $p16^{INK4a}$. Altered expression of retinoblastoma protein (pRb) is associated with non-Hodgkin's lymphoma, particularly centroblastic and Burkitt's lymphomas. pRb is expressed in normal B-cells and its regulatory phosphorylation pathway is activated in response to a variety of stimuli. Since human B-lymphoma-derived cell lines are often used as in vitro model systems to analyse the downstream effects of signal transduction, we examined the functional status of pRb in a panel of human B-cell lines. We identified eleven cell lines which express the hyperphosphorylated forms of pRb. Furthermore, we suggest that the pRb protein appears to be functional in these cell lines.

**Keywords:** retinoblastoma protein; cdkI; $p16^{INK4a}$; tumour suppressor; Burkitt's lymphoma; Epstein–Barr virus

Many human cancers harbour genetic changes that alter the function of the G1 cell cycle control check point (reviewed in Sherr and Roberts, 1995; Bartek et al, 1996; Hall and Peters, 1996; Palmero and Peters, 1996; Herwig and Strauss, 1997). The common outcome of these changes is the functional deregulation of the retinoblastoma protein (pRb). pRb directly interacts with and regulates the activity of a series of RNA polymerase I, II and III dependent transcription factors including members of the E2F family, MyoD and Elf-1 (Bartek et al, 1996; Sanchez and Dynlacht, 1996; Herwig and Strauss, 1997). The activity of pRb is regulated in turn by its phosphorylation status (Weinberg, 1995; Bartek et al, 1996; Herwig and Strauss, 1997; Mittnacht, 1998). In non-proliferating or quiescent cells, pRb is hypophosphorylated and active. However, in response to appropriate growth conditions, cells enter into the G1 phase of the cell division cycle, and cyclin-dependent kinase 4 (cdk4) and cdk6, in conjunction with members of the cyclin D family, and cdk2, in conjunction with cyclin E, phosphorylate pRb protein resulting in its inactivation. This releases both the pRb-associated transcription factors and the cell cycle control check point and allows cells to progress from G1 into S phase (Weinberg, 1995; Bartek et al, 1996; Herwig and Strauss, 1997; Mittnacht, 1998). Members of the $p16^{INK4a}$ and $p21^{CIP1}$ cdk-inhibitor families also influence the activity of pRb by directly inhibiting phosphorylation of pRb protein by the cdks (Bartek et al, 1996; Herwig and Strauss, 1997; Mittnacht, 1998). Thus, information from both positive and negative extracellular signals is integrated into a simple 'stop' or 'go' message at the G1 checkpoint by influencing the ability of the cyclin D–cdk and cyclin E–cdk complexes to phosphorylate pRb protein.

The retinoblastoma gene itself is a target for genetic alterations in many types of cancer (reviewed in Palmero and Peters, 1996). The resulting deletions and point mutations either destroy the coding capacity or else encode for non-functional versions of pRb protein (Cowell and Hogg, 1992; Weinberg, 1992). In cells that have sustained homozygous mutations to the retinoblastoma gene, pRb is constitutively inactive and, as a consequence, the pRb-dependent cell cycle check point cannot be imposed. Thus, mutation of the retinoblastoma gene can contribute to the deregulated proliferation of cancer cells.

The retinoblastoma gene is expressed in a cell cycle-dependent manner in normal human B-cells. As quiescent (Go) cells enter into the cell division cycle in response to relevant extracellular signals, two changes to pRb protein become apparent: the overall level of expression increases and the protein becomes phosphorylated on multiple sites (Sinclair et al, 1994, 1998; Hollyoake et al, 1995; Kempkes et al, 1995; Szekely et al, 1995; Cannell et al 1996). Altered expression of pRb has been described in primary samples derived from high grade non-Hodgkin's lymphoma (NHL) (Martinez et al, 1993) and expression is lost in 58% of centroblastic and Burkitt's lymphomas (Weide et al, 1994).

Cell lines derived from Burkitt's lymphomas and from Epstein–Barr virus (EBV) transformed human B-cells are frequently used as in vitro model systems to ask questions about the effects of extracellular signalling on the proliferation of B-cells (for example, Sinclair et al, 1993; Holder et al, 1993; Allday et al, 1995; Arvanitakis et al, 1995; MacDonald et al, 1996). Since pRb is involved in integrating a wide range of signal transduction pathways, it is important to determine whether or not established human B-cell lines express functional pRb protein.

In this report, the expression and the functional status of the retinoblastoma gene in 11 human B-cell lines are examined.

### MATERIALS AND METHODS

### Cell culture

The human B-lymphoid cell lines were maintained in RPMI supplemented with 15% (v/v) fetal calf serum, penicillin and streptomycin at between 2 and $8 \times 10^5$ cells per ml to ensure that they were in exponential growth. Ramos (Klein et al, 1975), DG75

*Received 13 May 1998*
*Revised 6 October 1998*
*Accepted 8 December 1998*

*Correspondence to:* AJ Sinclair

(Ben-Bassat et al, 1977), Akata (Takada, 1984), Rael (Klein et al, 1972), Mutu Cl 179 (Gregory et al, 1990), Mutu Cl 148 (Gregory et al, 1990) and Jijoye (Hinuma et al, 1967) were originally derived from Burkitt's lymphomas. LCL#3 (Sinclair et al, 1994), B95-8PF (Farrell et al, 1991) and IB4 (King et al, 1980) were established by immortalization of primary B-cells with the B95-8 strain of EBV. BJAB was originally derived from an EBV-negative B-lymphoma and does not harbour any of the c-*myc*/immunoglobulin translocations that are characteristic of Burkitt's lymphoma (Clements et al, 1975; Menezes et al, 1975). SAOS (Diller et al, 1990) and U2OS (Ponten and Saksela, 1967) were originally derived from osteosarcomas.

Quiescent primary B-cells were isolated from donated human blood using affinity purification with anti-CD19 coated magnetic beads (Dynal) as described previously (Sinclair et al, 1994; Sinclair and Farrell, 1995).

### Protein analysis

Total protein extracts were prepared from exponentially growing cell lines and from quiescent primary B-cells as described previously (Cannell et al, 1996). The protein concentrations were normalized after comparing the absorption at 280 nm. A total of 100 μg of each extract was fractionated on a sodium dodecyl sulphate (SDS)-polyacrylamide gel, transferred to polyvinylidene difluoride (PVDF) membrane (Immobilon-P) and processed as described previously (Cannell et al, 1996).

For the analysis of pRb protein, extracts were fractionated in a 10% gel and a monoclonal antibody used to detect the protein (14001A; Pharmingen). For the analysis of $p16^{INK4a}$ protein, extracts were fractionated in a 15% gel and a monoclonal antibody was used to detect the protein (DCS50; Lukas et al, 1995*a*). For the analysis of $p15^{INK4b}$ protein, extracts were fractionated in a 15% gel and a rabbit polyclonal antisera was used to detect the protein (K-18; Santa Cruz). For cdk4, proteins were fractionated in a 12.5% gel and a rabbit polyclonal antiserum was used to detect the protein (Bates et al, 1994). In each case an HRP-conjugated species-specific secondary antibody step was included and the signals were detected by enhanced chemiluminescence (ECL) after exposure to autoradiography (Amersham).

### Complex formation

Extracts were prepared by lysing cells in NP40 lysis buffer (50 mM Hepes pH 8.0; 1% (v/v) NP-40; 0.1% (v/v) Tween-20; 150 mM sodium chloride; 1 mM EDTA; 2.5 mM EGTA; 1 mM NaF; 10% (v/v) glycerol, 1 mM dithiothreitol; 'complete protease inhibitor' (Boehringer Mannheim)). Debris was removed from the extract by centrifugation (1000 rpm for 10 min in a Beckman J6B centrifuge). The extract was precleared by incubation with a mixture of protein A and protein G Sepharose for 45 min rocking at 4°C. Sepharose beads were removed by centrifugation (1000 rpm for 10 min in a Beckman J6B centrifuge). Cyclin D3 and any associated proteins were isolated from the precleared extract by precipitation with a cyclin D3 monoclonal antibody (DCS28; Welcker et al, 1996; Bartokova et al, 1998). For the IB4 extract only, a combination of DCS28 and a cyclin D2-specific antibody, DCS5.2 (Lukas et al, 1995*b*) was used to isolate both cyclin D2 and cyclin D3 associated proteins. A mock precipitation with control mouse antibodies (Sigma) was undertaken in parallel. The precipitated complexes were collected on a combination of Protein A and Protein G Sepharose beads (Sigma) and washed extensively with lysis buffer and then once with 50 mM Tris–HCl, pH 7.5. The proteins were then eluted from the beads by heating at 95°C for 5 min in 2 × PS buffer (0.12 M Tris pH 6.8; 4% (v/v) SDS; 20% (w/v) glycerol; 2% (v/v) β-mercaptoethanol; 0.01% (w/v) bromophenol blue) and fractionated on a 12.5% SDS-polycrylamide gel. Cdk4 protein in the complexes was detected by Western blot analysis using the rabbit polyclonal antiserum described above (Bates et al, 1994).

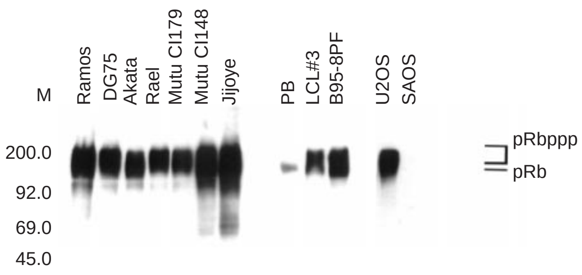

**Figure 1** Expression of pRb in B-lymphoid cells. Total protein extracts from a series of B-lymphoid cell lines and control cell lines were fractionated on a 10% SDS-acrylamide gel. The location of the molecular weight standards is indicated on the left (in kDa). The expression of pRb protein was determined by Western blot analysis. The migration of the hypophosphorylated and hyperphosphorylated forms of pRb are shown on the right

### Densitometric analysis

The relative level of $p16^{INK4a}$ protein detected by Western blot analysis was quantitated by densitometric analysis using Image Quant (Pharmacia) software.

### RESULTS

### pRb protein is expressed and regulated by phosphorylation in human B-lymphoid cell lines

The expression of pRb protein was surveyed in a series of well-characterized cell lines originally derived from Burkitt's lymphomas; Ramos, DG75, Akata, Rael, Mutu Cl 179, Mutu Cl 148 and Jijoye (Figure 1), and a B-cell lymphoma, BJAB (data not shown). In addition, three lymphoblastoid cell lines that had been previously immortalized with EBV in vitro; LCL#3 and B95-8PF (Figure 1) and IB4 (data not shown), were included in the analysis. The osteosarcoma cell lines, SAOS and U2OS, were analysed in parallel as controls. The SAOS cell line is known not to express detectable pRb protein, whereas the U2OS cell line expresses similar levels of pRb protein as proliferating primary human cells (Koh et al, 1995; Lukas et al, 1995*a*) (Figure 1). A single band of low intensity was observed in the sample of normal primary B-cells (PB). These cells were isolated from the peripheral circulation and the majority of the population were quiescent naive B-cells (Sinclair et al, 1994; Sinclair and Farrell, 1995). The single pRb band corresponded to the hypophosphorylated form of pRb which is characteristic of this cell type (Cannell et al, 1996). In contrast, all of the B-cell lines and the U2OS cell line displayed stronger signals with the pRb antibody. These appeared as a broad ladder or smear of bands, which corresponded to the multiply phosphorylated forms of pRb that are characteristic of proliferating cells (Buchkovich et al, 1989; Ludlow et al, 1989; Xu et al, 1989).

© Cancer Research Campaign 1999

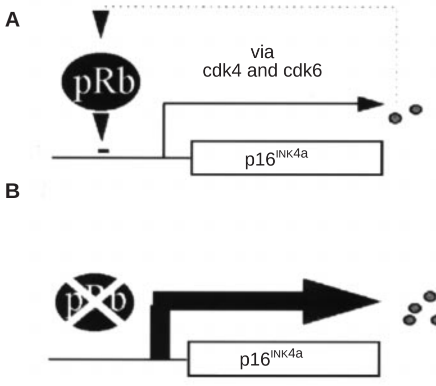

**Figure 2** Loss of functional pRb leads to increased p16$^{INK4a}$ expression. (**A**) The feedback loop linking pRb with the expression of p16$^{INK4a}$ is shown. (**B**) When pRb function is lost or compromised, the feedback loop is broken and p16$^{INK4a}$ expression is elevated

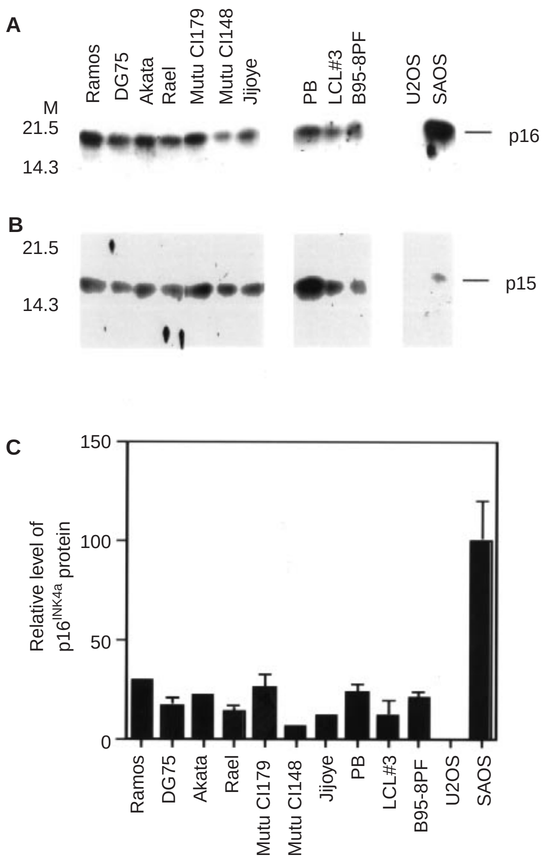

**Figure 3** Expression of p16$^{INK4a}$ and p15$^{INK4b}$ in B-lymphoid cells. (**A, B**) Total protein extracts from a series of B-lymphoid cell lines and control cell lines, as in Figure 1, were fractionated in duplicate on 15% SDS-acrylamide gels. The location of the molecular weight standards is indicated on the left (in kDa). The expression of p16$^{INK4a}$ protein (**A**) and p15$^{INK4b}$ protein (**B**) were determined by Western blot analysis. The migration of p16$^{INK4a}$ and p15$^{INK4b}$ are shown on the right. (**C**) The signals from p16$^{INK4a}$ protein were quantitated by densitometric analysis using Image Quant (Pharmacia) software. The levels of expression are plotted in arbitrary units with the level in SAOS cells set at 100

Thus, all 11 of the B-lymphoid cell lines contained equivalent levels of pRb protein, which is present in the hyperphosphorylated forms expected for proliferating cells.

### Analysis of pRb function in B-lymphoid cell lines

Although non-functional mutant forms of pRb do not generally exist in hyperphosphorylated forms (Scheffner et al, 1991; Templeton et al, 1991), the analysis of protein expression by Western blotting was not able to distinguish unequivocally between functional proteins and those that had sustained either small deletions within the coding sequence or point mutations which rendered them non-functional. We therefore sought further evidence to define whether the pRb proteins observed in this panel of B-cell lines were functional.

One consequence of the loss of pRb function in human cell lines is the increased expression of the cdkI gene p16$^{INK4a}$ (Serrano et al, 1993; Li et al, 1994; Otterson et al, 1994; Tam et al, 1994; Aagaard et al, 1995; He et al, 1995; Kelley et al, 1995; Kratzke et al, 1995; Lukas et al, 1995*a*; Musgrove et al, 1995; Parry et al, 1995; Shapiro et al, 1995*a,b*; Bartkova et al, 1996; Hara et al, 1996; Khleif et al, 1996; Itoh et al, 1997; Ruas and Peters, 1998). As illustrated in Figure 2, p16$^{INK4a}$ was expressed at basal levels in cells containing functional pRb. This was achieved by the repression of p16$^{INK4a}$ transcription by pRb protein. This repression formed part of a homeostatic feedback loop in which hypophosphorylated, active pRb repressed the transcription of p16$^{INK4a}$, maintaining basal levels of p16$^{INK4a}$ protein in the cell and allowing cdk4 and cdk6 to form active complexes with cyclin D and so phosphorylate pRb. As illustrated in Figure 2B, it is apparent that the functional inactivation of pRb disrupted this regulation, resulting in increased transcription of p16$^{INK4a}$ and the accumulation of high levels of p16$^{INK4a}$ protein within a cell. Indeed, it has been observed that human cell lines lacking functional pRb contain so much p16$^{INK4a}$ protein that no cyclin D–cdk4 or cyclin D–cdk6 complexes are formed (for example Serrano et al, 1993; Tam et al, 1994; Aagaard et al, 1995; Parry et al, 1995).

Thus, in a situation where apparently full-length pRb is expressed and the p16$^{INK4a}$ locus has not been deleted or silenced by methylation, the functional status of pRb can be questioned experimentally (i) by quantitating the relative level of p16$^{INK4a}$ protein within cells and (ii) by asking whether cyclin D–cdk4 and/or cyclin D–cdk6 complexes are present within the cell line.

In order to question whether pRb is functional in the panel of B-cell lines, the level of expression of p16$^{INK4a}$ protein was compared between the B-cell lines and the control cell lines. All of the samples were fractionated on the same gel and analysed in an identical fashion. As had been shown previously, the U2OS cell line did not express p16$^{INK4a}$ protein whereas the SAOS cell line, which did not express any pRb protein (Figure 1), contained characteristically high levels of p16$^{INK4a}$ protein (Lukas et al, 1995*a*) (Figure 3A). These two lines were included in the analysis as positive and negative controls. In comparison to the signal observed in SAOS cells, the normal B-cells (PB) and the B-lymphoid cell lines expressed p16$^{INK4a}$ protein at levels that were just detectable in this assay (Figure 3A). The expression of the related cdkI p15$^{INK4a}$, which is not regulated by pRb, was also compared between this panel of cell lines. p15$^{INK4b}$ did not show the same marked variation in expression (Figure 3B).

When the level of p16$^{INK4a}$ protein in the cell lines was quantitated, it became clear that there was some variation in the levels of

 © Cancer Research Campaign 1999

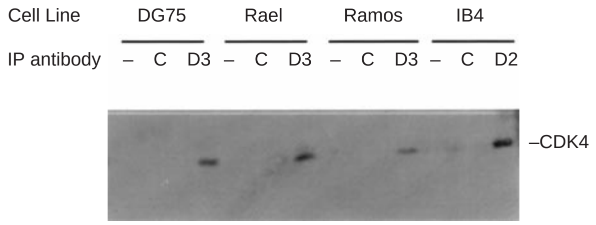

**Figure 4** Formation of cyclin D–cdk4 complexes in DG75 cells. NP-40 extracts were prepared from the indicated cell lines. After incubation with either anti-cyclin D3 alone (D3) or a combination of anti-cyclin D3 and anti-cyclin D2 primary antibodies (D2), or a control mouse IgG (C), or no antibody (–) the resulting complexes were isolated on a combination of protein A and protein G Sepharose beads and fractionated on a 12.5% SDS-acrylamide gel. The presence of cdk4 protein in the complexes was determined by Western blot analysis

$p16^{INK4a}$ protein between the B-cell lines (Figure 3C). However, the striking feature of the analysis was that none of the B-lymphoid cell lines expressed $p16^{INK4a}$ proteins at significantly higher levels than the normal primary B-cells, and none at levels comparable to that observed in the SAOS cell line. Thus, the level of $p16^{INK4a}$ protein within these B-lymphoid cell lines was consistent with the retention of functional pRb.

As a further test of the functional status of pRb within these cells, we analysed whether cyclin D–cdk4 complexes could be formed in four representative cell lines: an LCL (IB4), an EBV-positive Burkitt's lymphoma (Rael) and two EBV-negative Burkitt's lymphoma cell lines (DG75 and Ramos). These cell lines displayed characteristic differences in their expression of members of the cyclin D family; LCLs expressed predominantly cyclin D2, whereas the other cell lines expressed only cyclin D3 (Palmero et al, 1993; Pokrovskaja et al, 1996; Bartekova et al, 1998). In all four cell lines, isolation of complexes containing the relevant cyclin D protein resulted in the co-purification of cdk4 (Figure 4). Thus, the $p16^{INK4a}$ protein in these cell lines remained below the threshold required to disrupt cyclin D–cdk4 complexes, which further strengthened our general conclusion that pRb is functional in this panel of human B-lymphoid-derived cell lines.

### DISCUSSION

Although the incidence of genetic alteration to the retinoblastoma gene is high in non-Hodgkin's lymphomas (NHL) (Martinez et al, 1993; Weide et al, 1994), we have identified 11 human B-cell lines which express pRb protein. This suggests that at least one allele of the retinoblastoma gene is intact in the cell lines. Furthermore, since (i) the pRb/$p16^{INK4a}$ feedback loop does not appear to have been disrupted and (ii) cyclin D–cdk4 complexes exist in the cells, this suggests that the pRb protein is functional and so inactivating point mutations are unlikely to have occurred. These cell lines may therefore prove to be a useful background in which to investigate the consequences of experimentally disrupting pRb function in human B-cells.

It is intriguing that the published incidence of pRb lesions is higher in B-lymphoid malignancies than B-lymphoid cell lines. However, the discovery that the loss of functional pRb can promote apoptosis (Morganbesser et al, 1994; Pan and Griep, 1994) may suggest an explanation for this difference. Thus, a model can be formulated whereby B-cell tumours lacking functional pRb exist in vivo due to the presence of survival factors expressed by neighbouring cells; however, once they are removed from their microenvironment and placed into in vitro culture, they will not survive. Thus, it is likely that established B-lymphoid cell lines represent only a subset of the tumours.

The presence of slower migrating bands of pRb, as seen in Figure 1, shows that the pRb phosphorylation pathway is active in these cells. However, since components of this pathway are frequently subject to mutation in lymphoid malignancies it is worth considering whether other components of the pathway are likely to be compromised in these cell lines.

i. A chromosomal translocation, t(11;14) (Banks et al, 1992; Shivdasani et al, 1993; Harris et al, 1994), which juxtaposes the cyclin D1 gene with an immunoglobulin enhancer and thus drives the unscheduled expression of cyclin D1 in B-cells (reviewed in Hall and Peters, 1996), is associated with a subset of B-lymphomas (Mantle cell lymphomas). However, this translocation has not been found in Burkitt's lymphomas, which is consistent with the fact that no cyclin D1 protein or mRNA has been detected in an extensive group of cell lines derived from Burkitt's lymphomas or EBV immortalized B-cells (Palmero et al, 1993; Pokrovskaja et al, 1996).
ii. Homozygous deletion of the MTS1 (CDKN2A, $p16^{INK4a}$) locus is detected in a wide range of lymphoid malignancies (reviewed in Ruas and Peters, 1998). This results in a lack of expression of $p16^{INK4a}$ protein. As shown in Figure 3A, we were able to detect $p16^{INK4a}$ protein of the expected molecular weight in all of the B-cell lines, which implies that the locus has not been lost from this panel of cell lines.
iii. Point mutations within the $p16^{INK4a}$ coding sequence have also been observed in non-lymphoid malignancies (reviewed in Ruas and Peters, 1998). Although some of these changes are silent, some result in the expression of a protein which has lost its ability to inhibit cdk4 and cdk6. Although such point mutations are extremely rare in lymphoid malignancies (Ruas and Peters, 1998), we have not eliminated the possibility that they may be present in some of these B-cell lines.
iv. In addition, the molecular mechanisms by which $p16^{INK4a}$ expression can be altered includes silencing of the locus by site-specific methylation (Herman et al, 1995; Merlo et al, 1995; Otterson et al, 1995; Shapiro et al, 1995*a*,*b*). However, since we were able to detect expression of $p16^{INK4a}$ protein in these B-cell lines, this silencing mechanism is unlikely to play a significant role here.

The possibility remains that the $p16^{INK4a}$/pRb pathway is disrupted further downstream or bypassed in these cells. For example, it has been shown recently that elevated expression of either cyclin E or c-*myc* is able to promote G1 to S phase transition in the presence of hypophosphorylated, active pRb (Alevizopoulos et al, 1997; Leone et al, 1997; Lukas et al, 1997). Since all Burkitt's lymphoma cells express elevated levels of c-*myc* due to a translocation involving c-*myc* and one of the immunoglobulin loci (Rickinson and Keiff, 1996), this may reduce the selective pressure for mutations in the $p16^{INK4a}$/pRb pathway during the development of the tumour.

Thus, this panel of 11 cell lines, derived originally from B-cell malignancies (Burkitt's lymphomas) or Epstein–Barr virus transformation (lymphoblastoid cell lines) appear to express functional pRb and contain an active pRb phosphorylation pathway. In addition, this analysis demonstrates that the MTS1 locus is not silenced in the cell lines. From this, it can be concluded that neither the

© Cancer Research Campaign 1999

disruption of pRb function nor the silencing of MTS1 are absolutely required either for the generation of B-cell malignancies or for the subsequent outgrowth of B-cell lines.

### ACKNOWLEDGEMENTS

This work was supported by a project grant to AJS from the Leukaemia Research Fund. We thank D Lane, G Peters and J Bartek for antibodies, and M Rowe and K Takada for providing cell lines.

### REFERENCES

Aagaard L, Lukas J, Bartkova J, Kjerulff AA, Strauss M and Bartek J (1995) Aberrations of p16$^{INK4a}$ and retinoblastoma tumour-suppressor genes occur in distinct subsets of human cancer cell lines. *Int J Cancer* **61**: 115–120

Allday MJ, Sinclair AJ, Crawford DH and Farrell PJ (1995) Epstein-Barr virus efficiently immortalizes human B-cells without neutralising the function of p53. *EMBO J* **14**: 1382–1391

Alevizopoulos K, Vlavh J, Hennecke S and Amati B (1997) Cyclin E and c-myc proteins promote cell proliferation in the presence of p16INK4a and hypophosphorylated retinoblastoma family proteins *EMBO J* **16**: 5322–5333

Arvanitakis L, Yaseen N and Sharma S (1995) Latent membrane protein-1 induces cyclin D2 expression, pRb hyperphosphorylation, and loss of TGF-β-1-mediated growth-inhibition in EBV-positive B-cells. *J Immunol* **155**: 1047–1056

Banks PM, Chan J, Cleary ML, Delsol G, Dewolfpeeters C, Gatter K, Grogan TM, Harris NL, Isaacson PG, Jaffe ES, Mason D, Pileri S, Ralfkiaer E, Stein H and Warnke RA (1992) Mantle cell lymphoma – a proposal for unification of morphological, immunological, and molecular-data. *Am J Surg Pathol* **16**: 637–640

Bartek J, Bartekova J and Lukas J (1996) The retinoblastoma protein pathway and the restriction point. *Curr Opin Cell Biol* **8**: 805–8114

Bartekova J, Lukas J, Guldberg P, Alsner J, Kirkin AF, Zeuthen J and Bartek J (1996) The p16-cyclin-D cdk4-pRb pathway as a functional unit frequently altered in melanoma pathogenesis. *Cancer Res* **56**: 5475–5483

Bartekova J, Lukas J, Strauss M and Bartek J (1998) Cyclin D3: requirement for G1/S transition and high abundance in quiescent tissues suggest a dual role in proliferation and differentiation. *Oncogene* **17**: 1027–1037

Bates S, Bonetta L, MacAllan D, Parry D, Holder A, Dickson C and Peters G (1994) CDK6 (PLSTIRE) and CDK4 (PSK-J3) are a distinct subset of the cyclin-dependent kinases that associate with cyclin D1. *Oncogene* **9**: 71–79

Ben-bassat H, Goldblum N, Mitrani S, Goldblum T, Yoffey JM, Cohey MM, Bentwich Z, Ramot B, Klein E and Klein G (1977) Establishment in continuous culture of a new type of lymphocyte from a Burkitt-like malignant lymphoma (DG-75) *Int J Cancer* **19**: 27–33

Buchkovich K, Duffy LA and Harlow E (1989) The retinoblastoma protein is phosphorylated during specific phases of the cell-cycle. *Cell* **58**: 1097–1105

Cannell EJ, Farrell PJ and Sinclair AJ (1996) EBV exploits the normal cell pathway to regulate pRb activity during the immortalisation of primary B-cells. *Oncogene* **13**: 1413–1421

Clements GB, Klein G and Povey S (1975) Production by EBV infection of an EBNA-positive subline from an EBNA-negative human lymphoma cell line without EBV DNA. *Int J Cancer* **16**: 125–133

Cowell JK and Hogg A (1992) Genetics and cytogenetics of retinoblastoma. *Cancer Genet Cytogenet* **64**: 11–15

Diller L, Kassel J, Nelson CE, Gryka MA, Litwak G, Gebhardt M, Bressac B, Ozturk M, Baker SJ, Vogelstein B and Friend SH (1990) p53 functions as a cell-cycle control protein in osteosarcomas. *Mol Cell Biol* **10**: 5772–5781

Farrell PJ, Allan GJ, Shanahan F, Vousden KH and Crook T (1991) p53 is frequently mutated in Burkitt's lymphoma cell lines. *EMBO J* **10**: 2879–2887

Gregory CD, Rowe M and Rickinson AB (1990) Different Epstein–Barr virus-B cell interactions in phenotypically distinct clones of a Burkitt's lymphoma cell line. *J Gen Virol* **71**: 1481–1495

Hall M and Peters G (1996) Genetic alterations of cyclins, cyclin dependent kinases and cdk inhibitors in human cancer. *Adv Cancer Res* **68**: 68–108

Hara E, Smith R, Parry D, Tahara H, Steven S and Peters G (1996) Regulation of p16(CDKN2) expression and its implications for cell immortalization and senescence. *Mol Cell Biol* **16**: 859–867

Harris NL, Jaffe ES, Stein H, Banks PM, Chan J, Cleary ML, Delsol G, Dewolfpeeters C, Falini B, Gatter KC, Grogan TM, Isaacson PG, Knowles DM, Mason DY, Mullerhermelink HK, Pileri SA, Piris MA, Ralfkiaer E and Warnke RA (1994) A revised European–American classification of lymphoid neoplasms – a proposal from the international lymphoma study-group. *Blood* **84**: 1361–1392

He J, Olson JJ and James CD (1995) Lack of p16(INK4) or retinoblastoma protein (pRb), or amplification-associated overexpression of cdk4 is observed in distinct subsets of malignant glial tumors and cell-lines. *Cancer Res* **55**: 4833–4836

Herman JG, Merlo A, Mao L, Lapidus RG, Issa J, Davidson NE, Sidransky D and Baylin SB (1995) Inactivation of the CDKN2/p16/MTS1 gene is frequently associated with aberrant DNA methylation in all common human cancers. *Cancer Res* **55**: 4525–4530

Herwig S and Strauss M (1997) The retinoblastoma protein: a master regulator of cell cycle, differentiation and apoptosis. *Eur J Biochem* **246**: 581–601

Hinuma Y, Konn M, Yamaguchi J, Wudarski D, Blakeslee J and Grace J (1967) Immunofluorescence and herpes type virus particles in the P3HR-1 Burkitt lymphoma gene. *J Virol* **1**: 1045–1051

Holder MJ, Wang H, Milner AE, Casamayor M, Armitage R, Spriggs MK, Fanslow WC, MacLennan IC, Gregory CD and Gordon J (1993) Suppression of apoptosis in normal and neoplastic human B lymphocytes by CD40 ligand is independent of Bc1-2 induction. *Eur J Immunol* **23**: 2368–2371

Hollyoake M, Stühler A, Farrell P, Gordon J and Sinclair A (1995) The normal cell cycle activation programme is exploited during the infection of quiescent B-lymphocytes by Epstein-Barr virus. *Cancer Res* **55**: 4784–4787

Itoh N, Kakehi Y, Akao T, Kinoshita H, Okada Y and Yoshida O (1997) Concomitant presence of p16 cyclin-dependent kinase 4 and cyclin D cyclin-dependent kinase 4 complexes in LNCaP prostatic cancer cell line. *Jpn J Cancer Res* **88**: 229–233

Kelley MJ, Otterson GA, Kaye FJ, Popescu NC, Johnson BE and Dipaolo JA (1995) CDKN2 in HPV-positive and HPV-negative cervical-carcinoma cell-lines. *Int J Cancer* **63**: 226–230

Kempkes B, Spitkovsky D, Jansen-Durr P, Ellwart JW, Kremmer E, Delecluse HJ, Rottenberger C, Bornkamm GW and Hammerschmidt W (1995) B-cell proliferation and induction of early G1-regulating proteins by Epstein-Barr virus mutants conditional for EBNA2. *EMBO J* **14**: 88–96

Khleif SN, Degregori J, Yee CL, Otterson GA, Kaye FJ, Nevins JR and Howley PM (1996) Inhibition of cyclin D-cdk4/cdk6 activity is associated with an E2F-mediated induction of cyclin kinase inhibitor activity. *Proc Natl Acad Sci USA* **93**: 4350–4354

King W, Thomas-Powell AL, Raab-Traub N, Hawke M and Kieff E (1980) Epstein-Barr virus RNA. V. Viral RNA in a restringently infected, growth-transformed cell line. *J Virol* **36**: 506–518

Klein G, Ehlin-Hendrikson B and Schlossman S (1983) Induction of an activated b lymphocyte-associated surface moiety defined by the B2 monoclonal antibody by ebv conversion of an EBV-negative lymphoma line (Ramos): differential effect of transforming (B95-8) and nontransforming (P3HR-1) EBV substrains. *J Immunol* **130**: 1985–1989

Klein G, Giovanella B, Westman A, Stehlin J and Mumford D (1975) An EBV-genome-negative cell line established from an American Burkitt lymphoma; receptor characteristics EBV infectibility and permanent conversion into EBV-positive sublines by in vitro infection. *Intervirology* **5**: 319–334

Koh J, Enders GH, Dynlacht B and Harlow E (1995) Tumour derived p16 alleles encoding proteins defective in cell cycle inhibition. *Nature* **375**: 506–509

Kratzke RA, Otterson GA, Lincoln CE, Ewing S, Oie H, Geradts J and Kaye FJ (1995) Immunohistochemical analysis of the p16(ink4) cyclin-dependent kinase inhibitor in malignant mesothelioma. *J Natl Cancer Inst* **87**: 1870–1875

Leone G, DeGregori J, Sears R, Jakoi L and Nevins J (1997) Myc and ras collaborate in inducing accumulation of active cyclin E/cdk2 and E2F. *Nature* **387**: 422–426

Li Y, Nichols MA, Shay JW, and Xiong Y (1994) Transcriptional repression of the D-type cyclin dependent kinase inhibitor p16 by the retinoblastoma susceptibility gene product pRb. *Cancer Res* **54**: 6078–6082

Ludlow JW, Decaprio JA, Huang CM, Lee WH, Paucha E and Livingston DM (1989) SV40 large T-antigen binds preferentially to an underphosphorylated member of the retinoblastoma susceptibility gene-product family. *Cell* **56**: 57–65

Lukas J, Herzinger T, Hansen K, Moroni MC, Resnitzky D, Helin K, Reed SI and Bartek J (1997) Cyclin E-induced S-phase without activation of the pRb/E2F pathway. *Genes Dev* **11**: 1479–1492

Lukas J, Parry D, Aagaard L, Mann DJ, Bartokova J, Strauss M, Peters G and Bartek J (1995*a*) Retinoblastoma-protein dependent cell cycle inhibition by the tumour suppressor p16. *Nature* **375**: 503–506

 © Cancer Research Campaign 1999

Lukas J, Bartokova J, Welcker M, Peterson OW, Peters G, Strauss M and Bartek J (1995*b*) Cyclin D2 is a moderately oscillating nucleoprotein required for g1 phase progression in specific cell types. *Oncogene* **10**: 2125–2134

MacDonald I, Wang H, Grand R, Armitage R, Fanslow W, Gregory C and Gordon J (1996) Transforming growth factor β1 co-operates with anti-immunoglobulin for the induction of apoptosis in group I Burkitt's lymphomas cell lines. *Blood* **87**: 1147–1154

Martinez JC, Piris MA, Sanchezbeato M, Villuendas R, Orradre JL, Algara P, Sanchezverde L and Martinez P (1993) Retinoblastoma (Rb) gene-product expression in lymphomas – correlation with Ki67 growth fraction. *J Pathol* **169**: 405–412

Menezes J, Leibold W and Klein G (1975) Biological differences between Epstein-Barr virus (EBV) with regard to lymphocyte transforming ability, superinfection, and interference. *Exp Cell Res* **92**: 431–443

Merlo A, Herman JG, Mao L, Lee DJ, Gabrielson E, Burger PC, Baylin SB and Sidransky D (1995) 5′ CpG island methylation is associated with transcriptional silencing of the tumor-suppressor p16/CDKN2/MTS1 in human cancers. *Nat Med* **1**: 686–692

Mittnacht S (1998) Control of pRb phosphorylation. *Curr Opin Genet Dev* **8**: 21–27

Morganbesser SD, Williams BO, Jacks T and DePinho RA (1994) p53-dependent apoptosis produced by Rb-deficiency in the developing mouse lens. *Nature* **371**: 72–74

Musgrove EA, Lilischkis R, Cornish AL, Lee C, Setlur V, Seshadri R and Sutherland RL (1995) Expression of the cyclin-dependent kinase inhibitors p16(INK4), p15(INK4b) and p21(WAF1/CIP1) in human breast-cancer. *Int J Cancer* **63**: 584–591

Otterson GA, Kratzke RA, Coxon A, Kim YW and Kaye FJ (1994) Absence of p16INK4 protein is restricted to the subset of lung cancer lines that retains wildtype RB. *Oncogene* **9**: 3375–3378

Otterson GA, Khleif SN, Chen WD, Coxon AB and Kaye FJ (1995) Cdkn2 gene silencing in lung-cancer by dna hypermethylation and kinetics of p16(ink4) protein induction by 5-aza 2´ deoxycytidine. *Oncogene* **11**: 1211–1216

Palmero I and Peters G (1996) Perturbation of cell-cycle regulators in human cancer. *Cancer Surv* **27**: 351–367

Palmero I, Holder A, Sinclair AJ, Dickson C and Peters G (1993) Cyclins D1 and D2 are differentially expressed in human B-lymphoid cell lines. *Oncogene* **8**: 1049–1054

Pan H and Griep AE (1994) Altered cell cycle regulation in the lens of HPV-16 E6 or E7 transgenic mice: implications for tumour suppressor gene function in development. *Gene Dev* **8**: 1285–1299

Parry D, Bates S, Mann DJ and Peters G (1995) Lack of cyclin D-cdk complexes in Rb-negative cells correlates with high levels of p16 INK4/MTS-1 tumour suppressor gene product. *EMBO J* **14**: 503–511

Pokrovskaja K, Ehlinhenriksson B, Bartkova J, Bartek J, Scuderi R, Szekely L, Wiman KG and Klein G (1996) Phenotype-related differences in the expression of D-type cyclins in human B-cell-derived lines. *Cell Growth Differ* **7**: 1723–1732

Ponten J and Saksela E (1967) Spontaneous lymphoblast transformation of long-term cell cultures from human malignant lymphoma. *Int J Cancer* **2**: 434–447

Rickinson AB and Keiff E (1996) Epstein-Barr virus. In *Fields Virology*, Fields BN, Knipe DM, Howley PM (eds), pp. 3287–2446. Lippencot-Raven: Philadelphia

Ruas M and Peters G (1998) The p16INK4a/CDKN2A tumour suppressor and its relatives. *Biochem Biophys Acta* (in press)

Sanchez I and Dynlacht BD (1996) Transcriptional control of the cell-cycle. *Curr Opin Cell Biol* **8**: 318–324

Scheffner M, Münger K, Byrne JC and Howley PM (1991) The state of the p53 and retinoblastoma genes in human cervical carcinoma cell lines. *Proc Natl Acad Sci USA* **88**: 5523–5527

Serrano M, Hannon GJ and Beach D (1993) A new regulatory motif in cell-cycle control causing specific inhibition of cyclin D/CDK4. *Nature* **366**: 704–707

Shapiro GI, Edwards CD, Kobzik L, Godleski J, Richards W, Sugarbaker DJ and Rollins BJ (1995*a*) Reciprocal Rb inactivation and p16INK4 expression in primary lung cancers and cell lines. *Cancer Res* **55**: 505–509

Shapiro GI, Park JE, Edwards CD, Mao L, Merlo A, Sidransky D, Ewen ME and Rollins BJ (1995*b*) Multiple mechanisms of p16(INK4a) inactivation in non-small-cell lung-cancer cell-lines. *Cancer Res* **55**: 6200–6209

Sherr CJ and Roberts JM (1995) Inhibitors of mammalian G(1) cyclin-dependent kinases. *Genes Dev* **9**: 1149–1163

Shivdasani RA, Hess JL, Skarin AT and Pinkus GS (1993) Intermediate lymphocytic lymphoma – clinical and pathological features of a recently characterized subtype of non-Hodgkins-lymphoma. *J Clin Oncol* **11**: 802–811

Sinclair AJ and Farrell PJ (1995) Methods to study Epstein-Barr virus and p53 status in human cells. In *Methods in Molecular Genetics*, Aldolph KW (ed), pp. 89–100. Academic Press: Orlando

Sinclair AJ, Jacquemin MG, Brooks L, Shanahan F, Brimmell M, Rowe M and Farrell PJ (1993) Reduced signal transduction through glucocorticoid receptor in Burkitt's lymphoma cell lines. *Virology* **199**: 339–353

Sinclair AJ, Palmero I, Peters G and Farrell PJ (1994) EBNA-2 and EBNA-LP cooperate to cause G0 to G1 transition during immortalization of resting human B lymphocytes by Epstein–Barr virus. *EMBO J* **13**: 3321–3328

Sinclair AJ, Fenton M and Delikat D (1998) Interactions between EBV and the cell cycle control machinery. *Histol Histopathol* **13**: 461–467

Szekely L, Pokrovskaja K, Jiang W-Q, Selivanova G, Löwbeer M, Ringertz N, Wiman KG and Klein G (1995) Resting B-cells, EBV-infected blasts and established lymphoblastoid cell lines differ in their Rb, p53 and EBNA-5 expression patterns. *Oncogene* **10**: 1869–1874

Takada K (1984) Cross-linking of cell surface immunoglobulins induces Epstein-Barr virus in Burkitt lymphoma lines. *Int J Cancer* **33**: 27–32

Tam SW, Shay JW and Pagano M (1994) Differential expression and cell cycle regulation of the cyclin dependent kinases 4 inhibitor p16. *Cancer Res* **54**: 5816–5820

Templeton DJ, Park SH, Lanier L and Weinberg RA (1991) Nonfunctional mutants of the retinoblastoma protein are characterised by defects in phosphorylation, viral oncoprotein association and nuclear tethering. *Proc Natl Acad Sci (USA)* **88**: 3033–3037

Weide R, Tiemann M, Pfluger KH, Koppler H, Parvizl B, Wacker HH, Kreipe HH, Havemann K and Parwaresch MR (1994) Altered expression of the retinoblastoma gene-product in human high-grade non-Hodgkins-lymphomas. *Leukemia* **8**: 97–101

Weinberg RA (1992) The retinoblastoma gene and gene product. *Cancer Surv* **12**: 43–57

Weinberg RA (1995) The retinoblastoma protein and cell cycle control. *Cell* **81**: 323–330

Welcker M, Lukas J, Strauss M and Bartek J (1996) Enhanced protein stability: a novel mechanism of D-type cyclin over abundance identified in human sarcoma cells. *Oncogene* **13**: 419–425

Xu HJ, Hu SX, Hashimoto T, Takahashi R and Benedict WF (1989) The retinoblastoma susceptibility gene-product – a characteristic pattern in normal-cells and abnormal expression in malignant cells. *Oncogene* **4**: 807–812

© Cancer Research Campaign 1999
